# Cardiac Implantable Electronic Device Infections at a Tertiary Center in Southern Chile (2015–2021): A Retrospective Cohort Study

**DOI:** 10.3390/reports9030240

**Published:** 2026-07-24

**Authors:** Alban Landeros, Cheryld Mutel, Mauricio Soto, Luis Quiñiñir

**Affiliations:** 1 Department of Internal Medicine, Faculty of Medicine, Universidad de La Frontera, Araucanía Region, Temuco 4811230, Chile; albanlanderos@gmail.com (A.L.); cheryld.mutel@ufrontera.cl (C.M.); mauricio.soto@ufrontera.cl (M.S.); 2 Hospital de Pitrufquén, Araucanía Region, Pitrufquén 4920000, Chile; 3 Department of Cardiology, Hospital Dr. Hernán Henríquez Aravena, Manuel Montt 115, Araucanía Region, Temuco 4781151, Chile

**Keywords:** cardiac implantable electronic devices, device-related infections, pacemaker, implantable cardioverter-defibrillator, cardiac resynchronization therapy, staphylococcal infections, lead extraction, Latin America, Chile

## Abstract

Background/Objectives: Cardiac implantable electronic device (CIED) infections are infrequent but clinically significant, and Latin American—particularly Chilean—data remain scarce. We aimed to describe the clinical and microbiological profile, complications, mortality, and local infection burden of CIED infections at a tertiary center in southern Chile. Methods: This was a retrospective descriptive cohort study of all patients treated for CIED infection at Hospital Dr. Hernán Henríquez Aravena between January 2015 and December 2021. Crude per-procedure infection proportions were calculated using locally implanted devices (primary implants, generator replacements, and upgrades) as the denominator; because annual implant volumes and individual follow-up times were not retrievable, only exploratory approximate rates per 100 patient-years were derived under strong assumptions and were not used for formal comparison. Results: Fifty-four patients were included (77.8% men; mean age 69 ± 14 years). Predominant comorbidities were arterial hypertension (79.6%), heart failure (40.7%), atrial fibrillation (27.8%), and type 2 diabetes mellitus (24.1%). Pacemakers accounted for 59.3% of infections, and late-onset cases predominated (48.2%). The overall per-procedure infection proportion was 1.4% (95% confidence interval [CI] 1.1–1.9%) and was numerically higher for implantable cardioverter-defibrillators (ICDs; 5.5%) and cardiac resynchronization therapy (CRT) devices (4.3%) than for pacemakers (1.1%). Coagulase-negative Staphylococcus (43.2%) and *Staphylococcus aureus* (24.3%) were the leading isolates, although microbiological sampling was incomplete (available in 68.5%). Complete system extraction was attempted in all patients and achieved in all but one case; recurrence occurred in 9.3% and in-hospital mortality in 1.9%. Conclusions: The clinical and microbiological profile of CIED infections in this single-center southern Chilean cohort was broadly consistent with international series. Per-procedure proportions for ICDs and CRT devices were numerically higher than those for pacemakers, but the retrospective design, a procedure-based denominator including replacements and upgrades, and incomplete echocardiographic and microbiological workup preclude formal comparison with time-to-event registries; these device-specific findings should be regarded as exploratory and hypothesis-generating. The findings identify concrete, locally actionable targets: more systematic microbiological sampling, broader pre-procedural and diagnostic echocardiography (including transesophageal studies), and strengthened long-term follow-up of CIED carriers.

## 1. Introduction

Cardiac implantable electronic device (CIED) infections are an infrequent but clinically significant complication associated with substantial morbidity, prolonged hospitalization, and considerable healthcare costs. Large population-based and hospital cohorts have reported cumulative infection rates of approximately 1–2% over the device lifespan, with two- to four-fold higher figures for defibrillators and resynchronization systems than for conventional pacemakers [1,2,3,4]. This rising burden reflects the expanding global use of CIEDs, the older age of recipients, and the increasing comorbidity profile of contemporary candidates [4,5,6].

Recognized risk factors include chronic kidney disease (CKD), heart failure (HF), diabetes mellitus (DM), anticoagulant use, pocket hematoma, and procedural factors such as device revisions and generator or lead replacements [6,7,8,9]. CIED infections are conventionally classified by their temporal pattern as early (<30 days after implantation), delayed (30 days to 12 months), or late (>12 months), a distinction with pathophysiological and prognostic relevance [4,6].

Despite this growing body of evidence, data from Latin America remain limited and are largely derived from small institutional series focused on overall procedural complications rather than on infection-specific outcomes [10]. Published evidence from Chile is particularly scarce, hindering accurate estimation of the local burden and its epidemiological particularities. Characterizing the experience of high-volume referral centers in the region is therefore essential to inform regional benchmarks, identify modifiable risk factors, and guide preventive strategies.

The primary aim of this study was to describe the clinical and microbiological profile, complications, and mortality of CIED infections managed at Hospital Dr. Hernán Henríquez Aravena (HHHA; Temuco, Chile) between 2015 and 2021. Secondary objectives were to identify common risk factors, isolated microorganisms, and complications, and to estimate the local infection burden in devices implanted at the center during the same period.

## 2. Materials and Methods

### 2.1. Study Design and Setting

We conducted a retrospective descriptive cohort study including all consecutive patients treated for CIED infection at HHHA, a tertiary referral center for cardiac electrophysiology in southern Chile, between January 2015 and December 2021. The manuscript was prepared in accordance with the Strengthening the Reporting of Observational Studies in Epidemiology (STROBE) statement for cohort studies (see Supplementary Materials, Table S1).

### 2.2. Case Identification and Selection

Cases were identified through the institutional electrophysiology registry and systematic review of the hospital information system using International Classification of Diseases, 10th Revision (ICD-10) codes compatible with CIED infection. Data were extracted from physical and electronic medical records. Because the study began in 2015, prior to publication of the standardized European Heart Rhythm Association (EHRA) 2020 diagnostic criteria [6], formal application of the definite/possible/rejected EHRA classification was not feasible. The diagnosis of CIED infection was therefore established by the treating electrophysiologist on the basis of one or more of the following: (i) clinical signs of pocket infection (erythema, swelling, purulent discharge, dehiscence, or device exposure); (ii) microbiological isolation of an organism from pocket tissue, lead tip, or blood cultures in the context of clinical suspicion; or (iii) echocardiographic evidence of lead or valvular vegetation. Diagnoses were ascertained retrospectively from the clinical record as documented by the treating team. No independent second-reviewer adjudication or formal inter-rater reliability assessment was performed, and case definitions were not re-validated against a standardized checklist; because cases were diagnosed prospectively by several operators across the seven-year period, the consistency of case definition across operators and calendar years could not be verified retrospectively. This is acknowledged as a limitation (Section 4.5).

**Figure 1:**
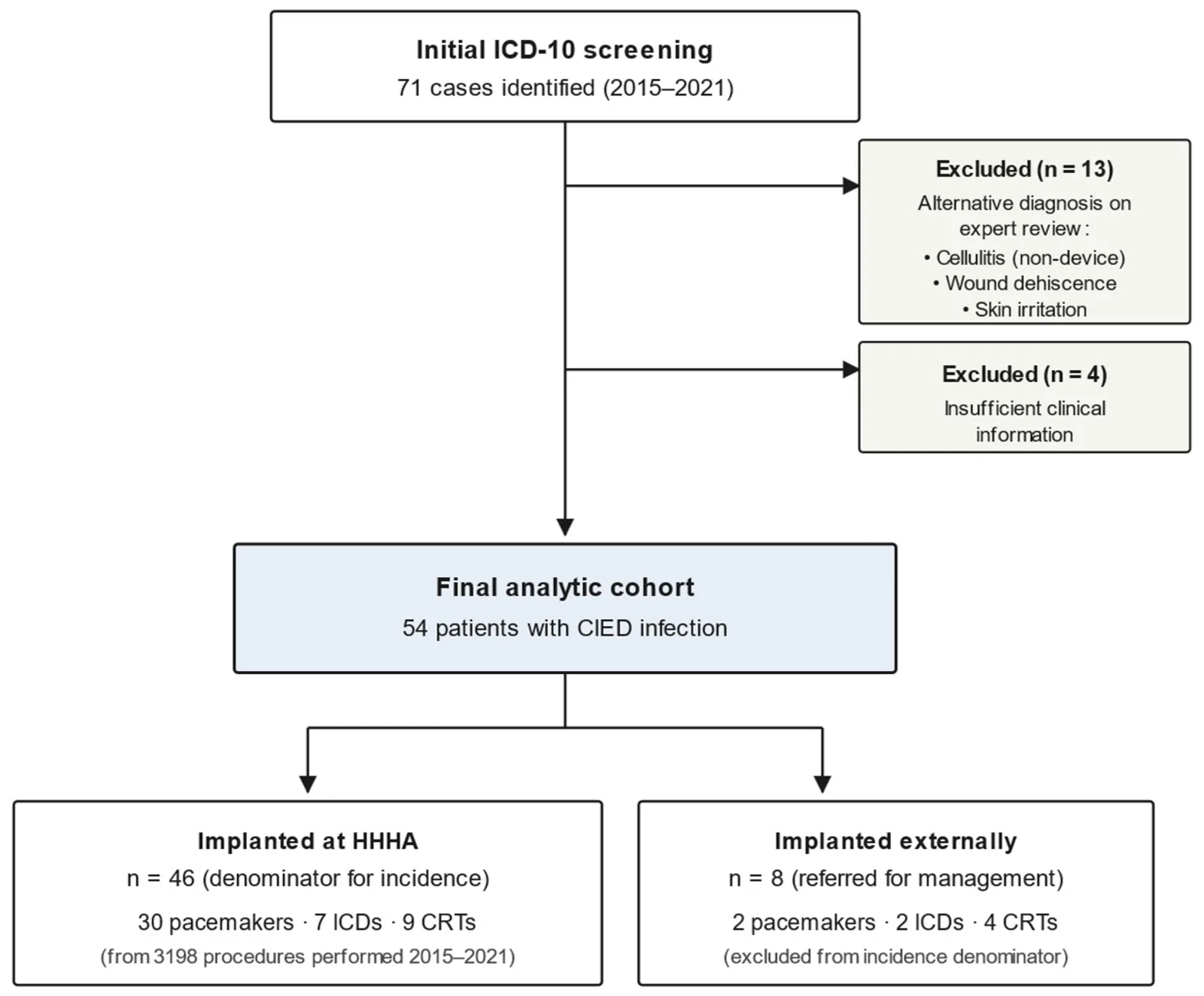
STROBE flow diagram of case selection. Of 71 cases initially identified through ICD-10 screening of the institutional electrophysiology registry and hospital information system, 13 were excluded after expert review (alternative diagnoses) and 4 were excluded due to insufficient clinical information, yielding 54 patients for analysis. CIED, cardiac implantable electronic device; HHHA, Hospital Dr. Hernán Henríquez Aravena; ICD-10, International Classification of Diseases, 10th Revision; STROBE, Strengthening the Reporting of Observational Studies in Epidemiology.

Of the 71 cases initially identified by ICD-10 screening, 13 were excluded after expert review because the final clinical diagnosis was not CIED infection (alternative diagnoses included cellulitis unrelated to the device, surgical wound dehiscence without infection, and superficial skin irritation), and 4 were excluded due to insufficient clinical information, yielding a final analytic cohort of 54 patients (Figure 1).

### 2.3. Operational Definitions and Local Burden Estimation

Recurrence was defined as any new device-related infection occurring within two years after reimplantation. Recurrences were ascertained from subsequent hospital admissions and outpatient electrophysiology visits documented in the institutional medical record system. No active systematic post-discharge surveillance was performed, and recurrences managed at external centers would not have been captured.

The local infection burden was calculated using only infections occurring in devices implanted at HHHA. During the study period, 3198 procedures were performed at the center—including primary implants, generator replacements, and upgrades—distributed as 2686 pacemakers (PMs), 127 implantable cardioverter-defibrillators (ICDs), 208 cardiac resynchronization therapy (CRT) devices, and 177 procedures whose device type could not be determined. Forty-six of these locally implanted devices subsequently developed confirmed infection.

The crude per-procedure infection proportion was calculated as the number of infections divided by the corresponding number of procedures. The denominator therefore corresponds to procedures rather than to unique patients and combines primary (de novo) implants with generator replacements and upgrades. Because replacement and upgrade procedures are recognized to carry a higher infection risk than de novo implantation, per-procedure proportions may overestimate the de novo infection risk; the exact proportion of replacement and upgrade procedures within the 3198-procedure denominator could not be retrieved from the institutional registry and is acknowledged as a limitation. Annual implant volumes and individual device follow-up times were likewise not retrievable. To provide an order-of-magnitude comparison only, exploratory approximate rates per 100 patient-years were derived under the strong and unverifiable assumptions of a uniform implant distribution across the seven-year period and a fixed mean per-device follow-up of 3.5 years. These exploratory approximate rates do not account for individual follow-up duration, censoring, competing events, or temporal growth in implant volume; they do not constitute formal incidence-density estimates and were not used as the basis for any statistical comparison with external registries.

### 2.4. Variables

The following variables were collected: age, sex, and comorbidities (arterial hypertension [AHT], type 2 diabetes mellitus [T2DM], atrial fibrillation [AF], CKD defined as stage V or requiring hemodialysis, HF, and use of anticoagulants or corticosteroids). Procedural and microbiological variables included antibiotic prophylaxis, type of implanted device, type of infection (local infection or cardiac device-related infective endocarditis [CDRIE]), isolated microorganism, and antimicrobial treatment (regimen and duration). Outcome variables comprised device extraction (technique and timing), associated complications (sepsis, osteomyelitis, embolism), and in-hospital mortality. Microbiological data were recorded as documented in the clinical record. Specimen types (blood cultures, pocket-tissue cultures, and lead-tip cultures) were not consistently distinguished in the source records and could not be analyzed separately.

### 2.5. Statistical Analysis

The statistical analysis was descriptive. Categorical variables are summarized as absolute frequencies and percentages. The distribution of continuous variables was assessed with the Shapiro–Wilk test; normally distributed variables are presented as mean ± standard deviation (SD), and non-normally distributed variables as median with interquartile range (IQR). Ninety-five percent confidence intervals (95% CIs) for proportions were computed using the Wilson score method for the overall and device-specific infection proportions, and using the Clopper–Pearson exact method for the microbiological proportions, where the smaller subgroup denominator (n = 37) made exact intervals preferable. No inferential analyses were performed. All analyses were conducted using Stata software, version 16 (StataCorp LLC, College Station, TX, USA). No generative artificial intelligence tools were used for data analysis.

### 2.6. Ethical Considerations

The study was conducted in accordance with the principles of the Declaration of Helsinki and was approved by the Scientific Ethics Committee of the Servicio de Salud Araucanía Sur (CEC SSAS; accredited by Resolución Exenta N° 2309584491 of 12 December 2023) under Official Letter (Oficio) N° 370 of 30 October 2024, with formal approval dated 28 October 2024. Given the retrospective design and the exclusive use of an anonymized institutional database extracted by an independent third party not involved in the analysis, the requirement for written informed consent was waived by the Ethics Committee.

## 3. Results

### 3.1. Demographics and Comorbidities

A total of 54 patients with CIED infection were included; 77.8% were male and 22.2% were female, with a mean age of 69 ± 14 years. Of these, 46 (85.2%) had been implanted at HHHA and 8 (14.8%) had been implanted at external centers and referred for management. AHT was present in 79.6%, T2DM in 24.1%, CKD stage V or on hemodialysis in 7.4%, AF in 27.8%, and HF in 40.7%. The median left ventricular ejection fraction (LVEF) was 46% (IQR 27–60). Anticoagulant use (vitamin K antagonists or direct oral anticoagulants) was documented in 18.5% of patients, recent device manipulation (any pocket or lead intervention within the 30 days preceding the diagnosis of infection) in 6 patients (11.1%), and previous use of a temporary pacing lead in 5 patients (9.3%). Detailed baseline characteristics are presented in Table 1.

### 3.2. Device Type, Timing, and Infection Proportion

Among the 54 infected devices, 32 were pacemakers (59.3%), 9 were implantable cardioverter-defibrillators (16.7%), and 13 were cardiac resynchronization therapy devices (24.1%) (Table 2) The 46 locally implanted infected devices (30 PMs, 7 ICDs, 9 CRTs) constituted the analytic denominator for incidence calculations, whereas the 8 externally implanted devices (2 PMs, 2 ICDs, 4 CRTs) were excluded from this denominator but retained in the descriptive analyses.

By temporal classification, 12.9% (n = 7) were early-onset, 38.8% (n = 21) were delayed-onset, and 48.2% (n = 26) were late-onset. The median time from implantation to diagnosis was 448 days (IQR 122–1252), with a mean of 858 ± 1003 days.

A total of 3198 CIEDs were implanted at HHHA during 2015–2021 (2686 PMs, 127 ICDs, 208 CRTs, and 177 unclassified procedures). Of these, 46 developed confirmed infection (30 PMs, 7 ICDs, and 9 CRTs), yielding an overall infection proportion of 1.4% (95% CI 1.1–1.9%). Device-specific infection proportions were 1.1% for PMs (95% CI 0.8–1.6%), 5.5% for ICDs (95% CI 2.7–10.9%), and 4.3% for CRTs (95% CI 2.3–8.0%) (Table 2). When restricted to infections occurring within the first post-implantation year (early plus delayed cases), the infection proportion was 1.6% for ICDs (2/127) and 2.4% for CRTs (5/208).

As an exploratory order-of-magnitude estimate only, approximate rates per 100 patient-years were derived under the assumptions described in the Methods (uniform implantation across the period and a fixed mean per-device follow-up of 3.5 years), yielding approximately 0.32, 1.57, and 1.24 per 100 patient-years for PMs, ICDs, and CRT devices, respectively. These figures are not formal incidence rates, rest on strong and unverifiable assumptions, and are not used here for quantitative comparison with registries employing time-to-event methodology; they are reported solely for transparency and hypothesis generation.

### 3.3. Type of Infection

Pocket infection was the most frequent clinical presentation (n = 36, 66.7%), followed by lead infection (n = 9, 16.7%) and a single case of infective endocarditis (1.9%); in 8 cases (14.8%), the anatomical type of infection could not be classified. The interval between implantation and diagnosis ranged from 2 to 4253 days (Table 3).

### 3.4. Microbiology

Microbiological samples were obtained from 37 patients (68.5%). Coagulase-negative *Staphylococcus* was the most frequently isolated organism (n = 16, 43.2%), of which 6 (37.5%) were oxacillin-resistant. *Staphylococcus aureus* was recovered in 9 patients (24.3%), all oxacillin-susceptible. Gram-negative bacilli were identified in 2 patients (5.4%) and *Enterococcus* spp. in 1 patient (2.7%); cultures were negative in 9 patients (24.3%). Microbiological findings are summarized in Table 4.

### 3.5. Echocardiographic Assessment

Echocardiographic assessment was incomplete in this cohort: 17 patients (31.5%) underwent transthoracic echocardiography (TTE), of whom only 2 (3.7% of the full cohort) also underwent transesophageal echocardiography (TEE); 27 patients (50.0%) had no echocardiographic study performed, and in 10 cases (18.5%), no information regarding echocardiography was retrievable. Because transesophageal echocardiography—the key examination for lead-related and valvular involvement—was performed in only 2 patients (3.7%) and half of the cohort had no echocardiographic study, cardiac device-related infective endocarditis could not be systematically assessed, and the single endocarditis case identified (1.9%) is very likely a substantial underestimate.

### 3.6. Antimicrobial Treatment

Antibiotic treatment information was available for 50 patients (92.6%). The most frequent empirical regimen was vancomycin monotherapy (n = 17, 34.0%), followed by cephalosporin monotherapy with cefazolin or cefadroxil (n = 15, 30.0%), vancomycin combined with a cephalosporin (n = 9, 18.0%), cloxacillin (n = 6, 12.0%), and other regimens (n = 3, 6.0%). The median duration of antibiotic therapy was 14 days (IQR 10–21), with a range of 0 to 65 days. The median hospital stay was 13 days (IQR 4–17).

### 3.7. Device Extraction and Reimplantation

Complete system extraction was attempted in all 54 patients. Simple manual traction was used in 38 cases (70.4%) and complex extraction techniques in 6 cases (11.1%), and the extraction technique was not documented in 10 cases (18.5%). In one patient, lead extraction was not feasible because of the unavailability of an advanced extraction device, with no subsequent complications. No surgical extractions were required, and no major peri-procedural complications (hemopericardium, pneumothorax, or peri-procedural death) were recorded. The median time from infection diagnosis to device extraction was 6.5 days (IQR 1.25–15.75; mean 12.1 ± 16.4 days; range 0–76 days). Reimplantation was performed in 41 of the 54 patients (75.9%).

### 3.8. Recurrence and Mortality

Five patients (9.3%) developed recurrent infection, with a mean age of 74 years. Four were pocket infections and one was a case of CDRIE. The mean time to recurrence was 9 months, distributed as: one case within the first month after implantation, three between 1 and 12 months, and one beyond the first year after definitive implantation. No fatal outcomes or major complications were documented in association with recurrence.

In-hospital mortality occurred in a single patient (1.9%, 95% CI 0.3–9.6%), corresponding to the only case of sepsis observed in the cohort. This patient had chronic kidney disease, multiple comorbidities, and a severe clinical course complicated by septic shock. No additional cases of sepsis, osteomyelitis, or embolic events were recorded.

## 4. Discussion

In this retrospective cohort from a tertiary referral center in southern Chile, the demographic and microbiological profile of CIED infections paralleled that of major international series [5,6], whereas crude per-procedure infection proportions for complex devices (ICDs and CRTs) appeared higher than those reported in large nationwide registries. The cohort also reveals locally relevant gaps in diagnostic workup, microbiological yield, and procedural surveillance that represent concrete opportunities for quality improvement.

### 4.1. Demographic and Microbiological Profile

The demographic profile observed in our cohort, with male predominance (77.8%) and a mean age of 69 years, mirrors that of previously published series, in which the higher comorbidity burden and broader indication for CIEDs among men may explain the observed pattern [5]. The prevalence of AHT, T2DM, HF, and AF is also consistent with the literature. The low proportion of patients with CKD (7.4%) is unexpected and likely reflects underreporting in the pre-digitization period rather than a true epidemiological difference [5,7,8].

Staphylococci predominated, with coagulase-negative *Staphylococcus* accounting for 43.2% of isolates and *Staphylococcus aureus* for 24.3%, with the majority being oxacillin-susceptible. These figures are in line with international series in which these two groups jointly represent 60% to 85% of CIED infections [4]. Microbiological documentation was nonetheless incomplete: 17 patients had no cultures obtained, and 24.3% of those cultured remained culture-negative. These gaps likely reflect early initiation of empirical antibiotic therapy before sampling, incomplete retrieval of prior microbiological records, and inconsistent collection of lead or deep-tissue specimens—patterns also reported in other cohorts. Because sampling was non-systematic and a substantial proportion of cultures were negative, the pathogen distribution is subject to selection bias, and the apparent predominance of staphylococci should be interpreted with caution, as fastidious, low-burden, or previously treated infections may be under-represented. Empirical regimens were dominated by vancomycin and cephalosporin monotherapy, with a median treatment duration of 14 days (IQR 10–21), matching the prevailing susceptibility profile. These findings support a systematic diagnostic protocol prioritizing pre-antibiotic blood cultures, routine deep-tissue sampling, and selective use of molecular diagnostics in culture-negative cases.

### 4.2. Device-Specific Infection Burden

Pacemakers accounted for the majority of infected devices (59.3%), reflecting their greater procedural volume rather than a higher per-device risk, in keeping with previous series [5]. The per-procedure infection proportion was numerically higher for more complex devices (5.5% for ICDs and 4.3% for CRTs versus 1.1% for pacemakers), values in the upper range of the published literature [4,5,9]. We deliberately refrain from drawing quantitative comparisons with registries that use time-to-event methodology—such as the Danish nationwide cohort [1]—because our denominator is procedure-based (including replacements and upgrades), complete implantation dates and individual follow-up times were unavailable, and the diagnostic workup (notably echocardiography) was incomplete. Any apparent excess in ICD/CRT infection relative to international registries should therefore be regarded as a hypothesis-generating signal rather than a confirmed difference, potentially influenced by the higher comorbidity burden of referred patients, a procedure-based denominator enriched in higher-risk replacements and upgrades, incomplete case ascertainment, and the small number of events. This signal nonetheless supports a local focus on procedural quality improvement for complex-device implantation, which is to be confirmed in prospective, person-time-based analyses.

### 4.3. Temporal Pattern and Prevention

The temporal distribution was dominated by late-onset (48.2%) and delayed-onset (38.8%) infections, which are typically attributed to transient bacteremia or distant infectious foci rather than to peri-implant contamination [6,8]. This pattern argues for long-term surveillance, comorbidity control, and prompt evaluation of any febrile or systemic infection in CIED carriers, particularly in elderly patients and those with multiple risk factors.

Regarding prevention, the elevated infection proportion observed for complex devices in our cohort highlights the relevance of contemporary preventive strategies. The Worldwide Randomized Antibiotic Envelope Infection Prevention (WRAP-IT) trial demonstrated that an absorbable antibacterial envelope reduced major CIED infections by approximately 40% in high-risk patients undergoing replacement, revision, or de novo CRT or ICD implantation [11]. Other measures recommended by current consensus documents include strict pre-procedural skin antisepsis, weight-adjusted intravenous antibiotic prophylaxis administered within 60 min of incision, meticulous hemostasis, axillary or cephalic venous access where feasible to minimize the number of cannulations, and timely treatment of pocket hematomas [6]. Leadless pacemakers, which eliminate both pocket and transvenous lead-related infections in selected single-chamber pacing indications, were not available at our institution during the study period; the leadless pacemaker program at HHHA was initiated in 2022, so these devices are not represented in the present cohort.

### 4.4. Extraction, Recurrence, and Mortality

Complete system extraction was attempted in all patients, in accordance with international recommendations that establish full hardware removal as the cornerstone of treatment to minimize recurrence and mortality [6,12]. Thirteen patients (24.1%) did not undergo subsequent reimplantation, suggesting that the original device indication warrants reassessment in the context of an infection. The specific reasons for non-reimplantation were not systematically captured but may include loss of clinical indication, clinician judgment regarding short life expectancy, patient preference, in-hospital death, or other causes; this question merits prospective investigation.

Recurrence occurred in 9.3% of patients, comparable to the 5–15% range typically reported in the literature, depending on cohort characteristics and recurrence definitions [4,12]. The mean age of patients with recurrence (74 years) was slightly higher than that of the overall cohort, which may signal an additional contribution from frailty, immunosenescence, or accumulated comorbidities. Three recurrences occurred in patients who had undergone single-stage explantation and reimplantation, a practice subsequently discouraged by international guidelines and discontinued at our center [6]. No fatal outcomes or major complications were associated with recurrence, an observation that likely reflects early antibiotic therapy, timely device extraction, and, in part, the limited sample size.

A single case of sepsis was documented and corresponded to the only in-hospital fatal outcome, yielding an in-hospital mortality of 1.9% (95% CI 0.3–9.6%). Although this figure appears at the lower end of the in-hospital mortality range reported in CIED infection cohorts [4,5], a single event provides limited statistical precision, and the confidence interval remains compatible with substantially higher mortality. The literature also reports much higher mortality at longer time horizons—between 8% and 20% at 1 year and up to 25–35% at 5 years in patients with device-related infective endocarditis [6,11]—which the in-hospital denominator used here cannot capture. No osteomyelitis or embolic events were observed in the cohort. The fatal case had CKD, multiple comorbidities, and prior use of a temporary pacing lead, factors that increase both the risk of infection and the risk of poor outcome, and that highlight the importance of careful patient selection and timely definitive device implantation. The absence of systematic post-discharge follow-up is therefore a significant limitation that precludes reliable estimation of medium- and long-term mortality in this cohort.

### 4.5. Limitations

This study has several limitations inherent to its retrospective, single-center design, and its findings should be interpreted as descriptive and hypothesis-generating rather than generalizable. First, because the EHRA 2020 diagnostic criteria were not available at study initiation, the case definition relied on the clinical, microbiological, and imaging-based judgment of the treating electrophysiologist; diagnoses were ascertained retrospectively, no independent second-reviewer adjudication or formal inter-rater reliability assessment was performed, and the consistency of case definition across operators and calendar years could not be verified, so a degree of misclassification cannot be excluded. Second, the denominator used for burden estimates corresponds to procedures rather than unique patients and combines primary implants with generator replacements and upgrades; because reintervention procedures carry a higher infection risk, the per-procedure proportions may overestimate de novo risk, and the exact proportion of replacement/upgrade procedures could not be retrieved from the registry. Third, annual implant volumes and individual follow-up times were not retrievable; consequently, formal incidence rates could not be calculated, and the per-100-patient-year figures are exploratory approximations derived under strong, unverifiable assumptions and were not used for statistical comparison with external registries. Fourth, the very low rate of TEE (3.7%) and the absence of any echocardiographic study in half of the patients constitute a critical diagnostic limitation that almost certainly led to marked underdiagnosis of CDRIE; the observed endocarditis proportion (1.9%) is implausibly low relative to the 10–23% reported internationally and reflects incomplete imaging rather than a true rate, so the true prevalence of CDRIE and of systemic infection cannot be reliably estimated and is likely underestimated, particularly because eight infections (14.8%) remained anatomically unclassifiable. Fifth, microbiological documentation was incomplete (cultures unavailable in 31.5% and culture-negative in 24.3% of those sampled), most likely owing to empirical antibiotics being started before sampling, incomplete record retrieval, and inconsistent deep-tissue/lead sampling; this non-systematic sampling introduces selection bias that may overstate the apparent predominance of staphylococci, and specimen types (blood versus pocket/lead cultures) were not consistently distinguished and could not be analyzed separately. Sixth, several established risk factors (body mass index, post-implant pocket hematoma, antibiotic prophylaxis regimen, corticosteroid use, vascular access route, total procedural time, and operator volume) were not captured systematically, limiting the ability to identify modifiable predictors. Seventh, no systematic post-discharge surveillance was performed, restricting mortality reporting to the in-hospital period and likely undercounting recurrences, particularly in the externally referred subgroup. Finally, the small number of events (one death, five recurrences, one endocarditis) yields wide confidence intervals and limits comparison with larger cohorts, and imaging confirmation of complete extraction was unavailable in 10 cases (18.5%). These limitations should be addressed in future prospective work incorporating standardized diagnostic criteria, complete person-time accounting, structured collection of risk factors, and active post-discharge surveillance.

## 5. Conclusions

In this seven-year, single-center cohort from southern Chile, CIED infections occurred with a crude per-procedure proportion of 1.4%, with numerically higher proportions for ICDs (5.5%) and CRT devices (4.3%). The overall clinical and microbiological profile was consistent with published series. Because of the retrospective single-center design, a procedure-based denominator that includes replacements and upgrades, and incomplete echocardiographic and microbiological evaluation, these device-specific proportions should be interpreted as exploratory and hypothesis-generating rather than as formal incidence estimates, and we refrain from concluding that local rates exceed those of international registries. System extraction was attempted in all patients and achieved in nearly all cases, with low in-hospital mortality (1.9%, 95% CI 0.3–9.6%) and a recurrence rate (9.3%) within the expected range, although the absence of long-term follow-up precludes assessment of medium- and long-term outcomes. The principal value of this study is descriptive: it contributes scarce real-world data from a Latin American—specifically Chilean—referral center and identifies concrete, locally actionable opportunities for quality improvement, including more systematic microbiological sampling, broader pre-procedural and diagnostic echocardiography (including transesophageal studies), structured documentation of antibiotic prophylaxis and pocket hematoma, and selective use of antibacterial envelopes in high-risk patients [6,11]. Prospective studies with standardized diagnostic criteria, complete person-time accounting, and active post-discharge surveillance will be needed to confirm these observations and to guide local preventive protocols.

## Figures and Tables

**Table 1:** Demographic characteristics and comorbidities of patients with CIED infection (n = 54).

Variable	Value
Age, years (mean ± SD)	69 ± 14
Male sex, n (%)	42 (77.8%)
Female sex, n (%)	12 (22.2%)
Arterial hypertension, n (%)	43 (79.6%)
Type 2 diabetes mellitus, n (%)	13 (24.1%)
Heart failure, n (%)	22 (40.7%)
LVEF, % (median, IQR)	46 (27–60)
Atrial fibrillation, n (%)	15 (27.8%)
Anticoagulant use, n (%)	10 (18.5%)
Chronic kidney disease (stage V or hemodialysis), n (%)	4 (7.4%)
Previous device manipulation, n (%)	6 (11.1%)
Previous temporary pacing lead, n (%)	5 (9.3%)

CIED, cardiac implantable electronic device; IQR, interquartile range; LVEF, left ventricular ejection fraction; SD, standard deviation.

**Table 2:** Crude per-procedure infection proportion of CIED infection by device type, 2015–2021.

Device Type	Total Implants (n)	Infections (n)	Infection Proportion (%)	95% CI
Pacemaker (PM)	2686	30	1.1	0.8–1.6
Implantable cardioverter-defibrillator (ICD)	127	7	5.5	2.7–10.9
Cardiac resynchronization therapy (CRT)	208	9	4.3	2.3–8.0
Unclassified	177	—	—	—
Total	3198	46	1.4	1.1–1.9

CI, confidence interval; CIED, cardiac implantable electronic device; CRT, cardiac resynchronization therapy; ICD, implantable cardioverter-defibrillator; PM, pacemaker. Infection proportion = (infections/total implants) × 100. The denominator includes primary implants, generator replacements, and upgrades. Unclassified procedures were excluded from device-specific estimates but retained in the overall denominator. First-year infection proportions (early plus delayed infections) were 1.6% for ICDs (2/127) and 2.4% for CRTs (5/208). Figures per 100 patient-years (0.32 for PMs, 1.57 for ICDs, 1.24 for CRTs) are exploratory approximate rates derived under strong assumptions (uniform implantation; 3.5-year mean follow-up); they are not formal incidence rates and are not used for comparison.

**Table 3:** Type of infection, timing, and interval from implantation to diagnosis.

	Value
Type of infection	
Pocket infection, n (%)	36 (66.7%)
Lead infection, n (%)	9 (16.7%)
Device-related infective endocarditis, n (%)	1 (1.9%)
Unclassifiable, n (%)	8 (14.8%)
Timing	
Early (<30 days), n (%)	7 (12.9%)
Delayed (30 days–12 months), n (%)	21 (38.8%)
Late (>12 months), n (%)	26 (48.2%)
Time from implantation to diagnosis	
Median (IQR), days	448 (122–1252)
Mean ± SD, days	858 ± 1003

IQR, interquartile range; SD, standard deviation.

**Table 4:** Microorganisms isolated from microbiological cultures (n = 37).

Microorganism	n (%)	95% CI
Coagulase-negative *Staphylococcus* (total)	16 (43.2%)	27.1–60.5
Oxacillin-susceptible	10 (27.0%)	14.6–43.1
Oxacillin-resistant	6 (16.2%)	6.2–31.3
*Staphylococcus aureus* (all oxacillin-susceptible)	9 (24.3%)	11.8–41.2
Gram-negative bacilli	2 (5.4%)	0.7–18.2
*Enterococcus* spp.	1 (2.7%)	0.1–14.2
Negative cultures	9 (24.3%)	11.8–41.2

CI, confidence interval (Clopper–Pearson exact method). Percentages calculated over the 37 patients with microbiological samples.

## Data Availability

The data presented in this study are available on request from the corresponding author. The data are not publicly available due to institutional data-protection policies and patient confidentiality considerations.

## Supplementary Materials

The following supporting information can be downloaded at: https://www.mdpi.com/article/10.3390/reports9030240/s1, Table S1: STROBE Statement Checklist for Cohort Studies.

## Abbreviations

AF	Atrial fibrillation
AHT	Arterial hypertension
CDRIE	Cardiac device-related infective endocarditis
CEC SSAS	Comité de Ética Científica, Servicio de Salud Araucanía Sur
CI	Confidence interval
CIED	Cardiac implantable electronic device
CKD	Chronic kidney disease
CRT	Cardiac resynchronization therapy
DM	Diabetes mellitus
EHRA	European Heart Rhythm Association
HF	Heart failure
HHHA	Hospital Dr. Hernán Henríquez Aravena
ICD	Implantable cardioverter-defibrillator
ICD-10	International Classification of Diseases, 10th Revision
IQR	Interquartile range
LVEF	Left ventricular ejection fraction
PM	Pacemaker
SD	Standard deviation
STROBE	Strengthening the Reporting of Observational Studies in Epidemiology
T2DM	Type 2 diabetes mellitus
TEE	Transesophageal echocardiography
TTE	Transthoracic echocardiography
WRAP-IT	Worldwide Randomized Antibiotic Envelope Infection Prevention (trial)
