# Supplementary material for: Cardiac Implantable Electronic Device Infections at a Tertiary Center in Southern Chile (2015–2021): A Retrospective Cohort Study
Source: Reports (MDPI). 2026 Jul 24;9(3):240. doi: 10.3390/reports9030240 (PMC13511300; doi:10.3390/reports9030240)
Supplement: Supplementary file 1 [file reports-09-00240-s001.zip › reports-4355761-supplementary.pdf]

Table S1. STROBE Statement Checklist for Cohort Studies

Manuscript: "Cardiac Implantable Electronic Device Infections at a Tertiary Center in Southern Chile (2015-2021): A Retrospective Cohort Study"

This checklist indicates where each STROBE cohort-study reporting item is addressed in the submitted manuscript. Page numbers refer to the submitted manuscript version prepared for Reports (MDPI).

Section / Topic	Item	STROBE recommendation	Reported in manuscript	Comments
Title and abstract	1(a)	Indicate the study design with a commonly used term in the title or abstract.	Title and Abstract, p. 1	The title states "Retrospective Cohort Study"; the abstract states "Retrospective descriptive cohort study."
Title and abstract	1(b)	Provide an informative and balanced summary of what was done and what was found.	Abstract, p. 1	Structured abstract includes background/objectives, methods, results, and conclusions.
Introduction: Background/rationale	2	Explain the scientific background and rationale for the investigation.	Introduction, pp. 1-2	Background describes CIED infection burden, risk factors, scarcity of Latin American/Chilean data, and need for regional benchmarks.
Introduction: Objectives	3	State specific objectives, including any prespecified hypotheses.	End of Introduction, p. 2	Primary and secondary objectives are clearly stated. No formal hypothesis is specified, consistent with a descriptive study.
Methods: Study design	4	Present key elements of study design early in the paper.	Methods 2.1, p. 2	Retrospective descriptive cohort study; setting and period are given.
Methods: Setting	5	Describe the setting, locations, relevant dates, periods of recruitment, exposure, follow-up, and data collection.	Methods 2.1-2.3, pp. 2-3	Tertiary referral center, study period 2015-2021, case identification, recurrence ascertainment, and lack of active surveillance are described.
Methods: Participants	6(a)	Give eligibility criteria and methods of participant selection.	Methods 2.2, pp. 2-3; Figure 1	All consecutive patients treated for CIED infection were included; exclusion after expert review and insufficient information are reported.
Methods: Participants	6(b)	For matched studies, give matching criteria and number of exposed and unexposed participants.	Not applicable	No matched design, exposed/unexposed comparison, or matching procedure was used.
Methods: Variables	7	Clearly define outcomes, exposures, predictors, confounders, and diagnostic criteria.	Methods 2.2-2.4, pp. 2-3	CIED infection diagnostic criteria, recurrence, local infection burden, comorbidities, microbiological variables, extraction, complications, and mortality are defined.
Methods: Data sources/measurement	8	For each variable of interest, give sources of data and details of methods of assessment.	Methods 2.2-2.4, pp. 2-3	Institutional electrophysiology registry, ICD-10 screening, hospital information system, physical/electronic records, and variable definitions are described.
Methods: Bias	9	Describe any efforts to address potential sources of bias.	Methods 2.2-2.3, pp. 2-3; Limitations, p. 10	Expert review excluded non-CIED infections; incidence denominator restricted to locally implanted devices; major sources of bias are explicitly acknowledged.
Methods: Study size	10	Explain how the study size was arrived at.	Methods 2.1-2.2, pp. 2-3; Results 3.1, p. 4	All consecutive eligible cases during 2015-2021 were included; 71 screened and 54 included.
Methods: Quantitative variables	11	Explain how quantitative variables were handled in the analyses and whether groupings were chosen.	Methods 2.3-2.5, pp. 3-4	Continuous variables are reported as mean +/- SD or median/IQR according to distribution; categorical variables as frequencies/percentages; infection proportions and approximate incidence rates are defined.

Section / Topic	Item	STROBE recommendation	Reported in manuscript	Comments
Methods: Statistical methods	12(a)	Describe all statistical methods, including those used to control for confounding.	Methods 2.5, pp. 3-4	Descriptive statistics, Shapiro-Wilk test, Wilson and Clopper-Pearson confidence intervals are described. No inferential analysis or confounding adjustment was performed.
Methods: Statistical methods	12(b)	Describe methods used to examine subgroups and interactions.	Not applicable	No subgroup interaction analyses were performed.
Methods: Statistical methods	12(c)	Explain how missing data were addressed.	Methods 2.2-2.4, pp. 2-3; Results 3.5-3.7, pp. 7-8; Limitations, p. 10	Insufficient clinical information led to exclusion of four cases; missing/unavailable echocardiography, microbiology, extraction technique, and risk-factor data are reported and discussed.
Methods: Statistical methods	12(d)	If applicable, explain how loss to follow-up was addressed.	Methods 2.3, p. 3; Limitations, p. 10	No active systematic post-discharge surveillance; recurrences outside the institution may not have been captured. This limitation is explicitly stated.
Methods: Statistical methods	12(e)	Describe any sensitivity analyses.	Not applicable	No sensitivity analyses were performed; study was descriptive.
Results: Participants	13(a)	Report numbers of individuals at each stage of the study.	Methods 2.2, p. 3; Figure 1	71 screened; 13 excluded after expert review; 4 excluded for insufficient information; 54 included.
Results: Participants	13(b)	Give reasons for non-participation at each stage.	Methods 2.2, p. 3; Figure 1	Alternative diagnoses and insufficient clinical information are stated as exclusion reasons.
Results: Participants	13(c)	Consider use of a flow diagram.	Figure 1	STROBE flow diagram is included.
Results: Descriptive data	14(a)	Give characteristics of study participants and information on exposures and potential confounders.	Results 3.1 and Table 1, pp. 4-5	Age, sex, comorbidities, LVEF, anticoagulant use, recent manipulation, and temporary pacing are reported.
Results: Descriptive data	14(b)	Indicate number of participants with missing data for each variable of interest.	Results 3.5-3.7, pp. 7-8; Limitations, p. 10	Missing echocardiography, antibiotic treatment availability, extraction technique not documented, absent cultures, and missing risk factors are reported where available.
Results: Descriptive data	14(c)	Summarize follow-up time.	Methods 2.3, p. 3; Results 3.8, p. 8	Recurrence defined within two years after reimplantation; mean time to recurrence is reported. No systematic long-term follow-up is acknowledged.
Results: Outcome data	15	Report numbers of outcome events or summary measures over time.	Results 3.2-3.8, pp. 5-8	Infection proportions, microbiological findings, extraction, recurrence, complications, and mortality are reported.
Results: Main results	16(a)	Give unadjusted estimates and, if applicable, confounder-adjusted estimates with precision.	Results 3.2, Table 2, pp. 5-6; Results 3.4, Table 4, pp. 6-7; Results 3.8, p. 8	Unadjusted infection proportions, approximate incidence rates, microbiological proportions, recurrence, and mortality with confidence intervals where reported are provided.
Results: Main results	16(b)	Report category boundaries when continuous variables were categorized.	Methods 2.2-2.4, pp. 2-3; Results 3.3, Table 3, p. 6	Temporal categories are explicitly defined: early <30 days, delayed 30 days-12 months, late >12 months. CKD definition is given.
Results: Main results	16(c)	If relevant, translate estimates of relative risk into absolute risk for a meaningful time period.	Not applicable	No relative risk estimates were calculated; the study reports absolute infection proportions and approximate rates.
Results: Other analyses	17	Report other analyses, including subgroup or sensitivity analyses.	Not applicable	No additional subgroup, interaction, or sensitivity analyses were conducted.
Discussion: Key results	18	Summarize key results with reference to study objectives.	Discussion opening paragraph, p. 8	Key demographic, microbiological, device-specific infection burden, and quality-improvement findings are summarized.

Table S1 - STROBE Checklist

Section / Topic	Item	STROBE recommendation	Reported in manuscript	Comments
Discussion: Limitations	19	Discuss limitations, considering potential sources of bias or imprecision.	Discussion 4.5, p. 10	Retrospective single-center design, non-standardized case definition, denominator limitations, missing risk factors, low TEE use, incomplete microbiology, limited follow-up, small event numbers, and incomplete imaging confirmation are discussed.
Discussion: Interpretation	20	Give a cautious overall interpretation considering objectives, limitations, multiplicity of analyses, similar studies, and relevant evidence.	Discussion 4.1-4.5, pp. 8-10; Conclusions, pp. 10-11	Interpretation compares findings with international cohorts and emphasizes cautious reading of approximate rates and diagnostic limitations.
Discussion: Generalizability	21	Discuss the generalizability of the study results.	Introduction, p. 2; Discussion 4.2 and 4.5, pp. 8-10; Conclusions, pp. 10-11	The manuscript frames findings as a single-center experience from southern Chile and emphasizes regional benchmarking rather than universal generalization.
Other information: Funding	22	Give the source of funding and role of funders.	Declarations: Funding, p. 12	The manuscript states that no external funding was received.

Abbreviations: CDRIE, cardiac device-related infective endocarditis; CIED, cardiac implantable electronic device; CRT, cardiac resynchronization therapy; HHHA, Hospital Dr. Hernán Henríquez Aravena; ICD, implantable cardioverter-defibrillator; IQR, interquartile range; LVEF, left ventricular ejection fraction; PM, pacemaker; SD, standard deviation; STROBE, Strengthening the Reporting of Observational Studies in Epidemiology; TEE, transesophageal echocardiography.
